# Resident Corneal Cells Communicate with Neutrophils Leading to the Production of IP-10 during the Primary Inflammatory Response to HSV-1 Infection

**DOI:** 10.1155/2012/810359

**Published:** 2012-03-15

**Authors:** S. J. Molesworth-Kenyon, N. Popham, A. Milam, J. E. Oakes, R. N. Lausch

**Affiliations:** ^1^Department of Biology, University of West Georgia, Carrollton, GA 30118, USA; ^2^Department of Microbiology and Immunology, University of South Alabama, Mobile, AL 36688, USA

## Abstract

In this study we show that murine and human neutrophils are capable of secreting IP-10 in response to communication from the HSV-1 infected cornea and that they do so in a time frame associated with the recruitment of CD8^+^ T cells and CXCR3-expressing cells. Cellular markers were used to establish that neutrophil influx corresponded in time to peak IP-10 production, and cellular depletion confirmed neutrophils to be a significant source of IP-10 during HSV-1 corneal infection in mice. A novel *ex vivo* model for human corneal tissue infection with HSV-1 was used to confirm that cells resident in the cornea are also capable of stimulating neutrophils to secrete IP-10. Our results support the hypothesis that neutrophils play a key role in T-cell recruitment and control of viral replication during HSV-1 corneal infection through the production of the T-cell recruiting chemokine IP-10.

## 1. Introduction

 Herpes simplex virus-type 1 (HSV-1) infection of the human cornea can lead to a damaging inflammatory response known as herpes stromal keratitis (HSK). According to the National Eye Institute, 50,000 new and recurring HSV-1 ocular infections are reported annually, and HSK is the leading cause of infectious blindness in the United States [1]. During physical trauma or HSV-1 infection, cells resident in the cornea initiate an immune response through the production of proinflammatory mediators such as IL-1*α*, IL-6, and the neutrophil chemoattractant CXCL8 (homologous to MIP-2 in the mouse) [2–5]. Mice also exhibit an inflammatory response to HSV-1 infection of the cornea which is marked by neutrophil infiltration at days 2 and 9 after infection (p.i.) [6–9]. In the mouse, CD8^+^ T cells are required for viral clearance around day 8 after infection, while the CD4^+^ T-cell subset has been described as having a role in the development of HSK and CD8^+^ cell regulation after viral clearance has occurred [8, 10, 11].

 The role of the neutrophil in the immune response to HSV-1 infection has not yet been fully explored. However, neutrophil depletion studies have demonstrated that in the absence of neutrophils CD8^+^, T-cell levels are reduced and viral clearance is limited leading to more severe disease development [12, 13]. Divito and Hendricks [14] reported that neutrophil accumulation in addition to CD4^+^ T-cell infiltration was necessary for HSK development. Previous studies reported from our lab and others indicate a role for neutrophils in the secretion of T-cell-recruiting chemokines IP-10 (Interferon gamma-induced protein 10 or CXCL10) and MIG (Monokine induced by gamma interferon or CXCL9) [15, 16]. It is known that T lymphocytes express the receptor CXCR3 and may be recruited by the ligands IP-10 and MIG and that antibody neutralization of IP-10 results in increased viral titers during HSV-1 corneal infection [17, 18]. We previously used a model for delayed type hypersensitivity (DTH), a secondary immune response to HSV-1 antigen in the skin of mice, to demonstrate that the neutrophil acts as a source for both IP-10 and MIG in the model. In the absence of neutrophil recruitment, T lymphocyte numbers were reduced during the DTH response [15, 19]. Thus, although the neutrophil has traditionally been described as merely a phagocytic cell, there is now increasing evidence to support the hypothesis that it may also function to bridge the response between the innate and adaptive immune system.

 In the study presented here, we hypothesize that neutrophils play a role in T-cell recruitment into the HSV-1 infected cornea through the production of IP-10. We describe the use of a model for the inflammatory response to a primary HSV-1 infection of the murine cornea and a novel *ex vivo* system for the study of primary HSV-1 infection in human corneas. Data is presented from experiments designed to investigate the production kinetics of chemokine IP-10 and its receptors during infection. We also demonstrate the effect of cellular depletion of neutrophils, natural killer cells, and CD4^+^ T cells on the level of IP-10 production and provide evidence for secretion of IP-10 by neutrophils, *in vitro* and *in vivo*, in both murine and human corneal tissue infected by HSV-1.

## 2. Materials and Methods

### 2.1. Mice

 Six-week-old female C57Bl/6 mice were obtained from Jackson Laboratories (Bar Harbour, ME). All animals were cared for in accordance with federal, state, and local regulations.

### 2.2. Antibodies and Reagents

 Anti-mouse granulocyte mAb RB6-8C5 was used for the depletion of neutrophils and was a gift from Robert Coffman (DNAX Research Institute, Palo Alto, CA). Anti-mouse CD4 hybridoma clone GK1.5 was obtained from the American Type Culture Collection (ATCC, Manassas, VA). Antibodies were prepared from hybridoma lines as previously described [13]. A combination of rabbit antiasialo GM1 (Wako Pure Chemical Industries, Ltd., Richmond, VA) and mAb NK1.1 (BD Biosciences, San Diego, CA) were used for NK cell depletion. Recombinant mouse IL-1*α* and IFN-*γ*) were purchased from R&D Systems (Minneapolis, MN.).

### 2.3. Topical and Intracorneal HSV-1 Infection of Murine Corneas

 For experiments investigating the protein kinetics of IP-10, infections were achieved topically by applying 2 × 10^5^ PFU/2 *μ*L HSV-1 strain RE on the scarified cornea of the mice. For mRNA investigation and cellular depletion studies, infection was performed using an intrastromal route to reduce intragroup variability. In brief, a pilot hole was formed through the epithelium of the cornea, with a 30-gauge disposable needle, through which a 32-gauge, 30 cm needle attached to a repeating dispenser (Hamilton, Reno, NV) was then inserted. 1 × 10^4^ or 1 × 10^5^ PFU HSV-1 RE (as indicated in the figure legend) was injected intrastromally in a volume of 1 *μ*L.

### 2.4. Corneal Opacity Scores

 Eyes were monitored for corneal opacity by visual observation under the dissecting microscope and graded as follows: 0 = clear cornea, 1 = slight corneal haze, 2 = moderate corneal opacity, 3 = severe corneal opacity but iris visible, 4 = severe corneal opacity with iris obscured, and 5 = necrotizing stromal keratitis.

### 2.5. Virus Titration

 Corneas were titrated for infectious virus on vero cell monolayers in a standard 48 h plaque assay.

### 2.6. mRNA Extraction and Real-Time PCR Analysis

 In experiments where upregulation of mRNA levels was investigated, corneas from 5 mice of the same experimental group were excised and pooled. The pooled corneas were homogenized for 30 s in 0.5 mL of RNAwiz (Ambion Inc. Austin, TX) and total RNA was then extracted following the manufacturers protocol. Contaminating genomic DNA was removed by DNase treatment using DNA free (Ambion Inc., Austin, TX), while total RNA purity and quantity were determined using the Bio-Rad SmartSpec 3000 spectrophotometer (Hercules, CA). Ambion Message Sensor RT (reverse transcriptase) kit was used to convert 0.5 *μ*g total RNA to cDNA for real-time PCR analysis. Bio-Rad iQ SYBR Green Supermix in a 96-well plate format was used for analysis with a Bio-Rad iCycler IQ system. GAPD mRNA levels were used to normalize template loading variations and negative RT reactions were performed to ensure the absence of genomic contamination in the samples. mRNA levels are reported as a fold increase over expression levels in untreated control corneas.

### 2.7. Murine Corneal Fibroblast Cell Culture

 Sixteen corneas from 8 uninfected mice were excised, pooled, and incubated at 37°C, 5% CO_2_ in 5 mM EDTA/PBS for 20 min. After this time, epithelial sheets were removed with forceps under the dissecting microscope. The stromal layers of each cornea were minced and incubated in 1500 U/mL of collagenase type I (Sigma) at 37°C, 5% CO_2_, for 1 h. The digested stromal layers were washed repeatedly in DMEM containing 20% FBS. Resultant cells were cultured in a T25 tissue culture flask in minimal volume of DMEM + 20% FBS medium. After 1 week of culture, cells were trypsinized and redistributed to the same flask to avoid clumping. At 90% confluency cells were passaged to a 12-well tissue culture plate at a density of 4 × 10^4^ cells/well. Cells were serum starved for 3 days prior to stimulation for 24 h with IFN-*γ* or IL-1*α*. Each stimulation was performed in triplicate and chemokine production was quantitated by ELISA.

### 2.8. Murine Neutrophil Isolation and Stimulation

 Neutrophils were isolated from the bone marrow of mice as previously described [20]. In brief, bone marrow cells extracted from the hind limbs of mice were gradient purified over Histopaque 1119 and 1077 (Sigma, St. Louis, MO). The enriched neutrophil band was washed in medium and treated with red blood cell lysis buffer (Sigma). Contaminating monocytes were depleted by adherence to a polystyrene culture plate. Neutrophil purity was established to be consistently >99%, by HEMA3 (Biochemical Sciences) staining of cytospin slides. For *in vitro* stimulation assays, 1 × 10^6^ neutrophils in 0.5 mL medium were placed in triplicate in 24-well tissue culture plates (Corning, New York, NY) previously coated with newborn calf serum (NCS). Neutrophils were stimulated for 8 h at 37°C, 5% CO_2_, with IL-1*α*, IFN-*γ*, or HSV-1. Medium alone was used as a negative control. Supernatants were collected, clarified, and assayed by ELISA for protein levels.

### 2.9. Chemokine Protein Assay

 Corneas were excised and cleaned of limbal tissue at the indicated times and processed by homogenization (30 seconds in a tissue tearer; Biospec Products, Bartlesville, OK) and sonication (15 s) in a total of 0.5 mL RPMI + 10% NCS unless stated otherwise. Corneal samples were clarified by centrifugation at 150 × g for 10 min and supernatants were analyzed by ELISA. In cell studies, supernatants were collected and assayed for secreted chemokine levels by ELISA. Murine IP-10 and MIP-2 ELISA assay kit sensitivities were 2.2 and 1.2 pg/mL, respectively, and the human IP-10 assay kit had a mean minimal detectable dose of 1.67 pg/mL. All kits were obtained from R&D systems (Minneapolis, MN.).

### 2.10. *In Vivo* Cellular Depletions

 To achieve *in vivo* depletion of cellular subsets, 0.5 mg of mAb RB6-8C5 (neutrophil depletion), 0.5 mg GK1.5 (CD4^+^ T-cell depletion), or 1 mg antiasialo GMI admixed with 0.1 mg NK1.1 (NK cell depletion) were administered by intraperitoneal injection to mice 3 h prior to HSV-1 challenge [13, 21–23]. Mice were challenged by intrastromal injection of 1 × 10^5^ PFU of HSV-1 and corneas excised at the times indicated. Depletion of neutrophils was confirmed by differential staining of blood smears. FACS analysis of spleen cells was performed using GK1.5 and NK1.1 to quantify CD4^+^ T-cell and NK-cell depletion levels, respectively. Excised corneas were processed by homogenization and sonication to produce a lysate for chemokine analysis by quantitative ELISA.

### 2.11. Human Corneal Tissue Infection

 Human corneas were obtained from the Georgia Eye Bank, Inc. EBAA (Atlanta, GA). All donors were screened and found to be nonreactive for HIV and Hepatitis. Corneas were released for research upon failure to meet transplantation criteria.

 Four, 4 mm corneal buttons were punched from each cornea using an arch punch (C.S. Osborne Tools). A minimum of three donors were used per experiment. Corneal buttons were placed in 24-well tissue culture plates containing 0.5 mL serum free RPMI 1640 media for incubation at 37°C, 5% CO_2_. Corneal button surfaces were scarified using an 18-gauge needle to mimic the murine topical infection protocol. For HSV-1 infected samples 1 × 10^6^ PFU of virus was added to the corneal button in the well. Infected and uninfected corneas were then incubated in the presence or absence of purified human neutrophils, 1 × 10^6^ neutrophils/well at 37°C, 5% CO_2_, for 24 h. Media, cells, and corneal buttons were removed and processed by sonication (30 s). Quantitation of human IP-10 chemokine levels was performed by ELISA. Levels of the chemokine CXCL8 were monitored as a marker of inflammation. This model permits the study of the interaction between resident corneal cells, neutrophils, and virus in the absence of other cell types which could be recruited to the site of inflammation *in vivo*.

### 2.12. Human Neutrophil Isolation

 Neutrophils were obtained from freshly donated venous blood. Gradient purification was achieved following a protocol equivalent to that described above for the murine neutrophils.

### 2.13. Statistical Analysis

 Student's *t* test was performed to determine significant differences between experimental and control groups which each contained a minimum of three mice or three human corneal donors. A value of *P* < 0.05 was considered significant. A nonparametric test was performed on clinical samples where indicated in the figure legend. A representative experiment is shown in each figure with experiments having been performed multiple times.

## 3. Results

### 3.1. CXCR3 mRNA Is Detected in the Infected Murine Cornea at 6 Days Post Infection

**Figure 1:**
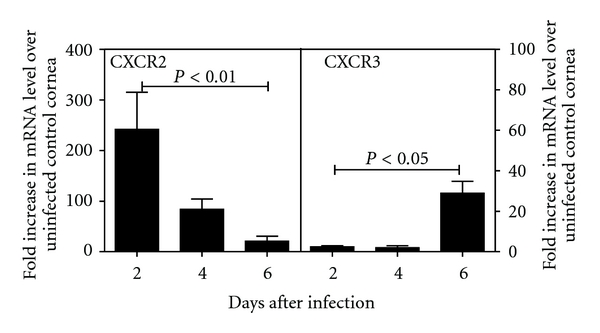
CXCR2 and CXCR3 mRNA are detected in the infected cornea at 2 and 6 days post infection respectively. Mice (*n* = 3) were challenged by intrastromal injection with 1 × 10^4^ PFU HSV-1 and corneas were harvested on the days indicated post infection. Control mice were uninfected and corneas from this group represented the level of constitutive mRNA production for each receptor. Total RNA was isolated from each group and converted to cDNA which was analyzed by real-time PCR. Upregulation in mRNA for each receptor is reported as the fold increase over production in uninfected control corneas.

 It has been shown previously in the murine model for HSV-1 corneal infection that the virus is cleared within 8 days due to the recruitment of CD8^+^ T lymphocytes [24]. With the focus of this study being the T-cell recruiting chemokine IP-10, it was necessary to establish that the T lymphocytes recruited to the HSV-1 infected cornea were expressing the CXCR3 receptor for IP-10. Mice were infected by intrastromal injection of 1 × 10^4^ PFU HSV-1 and corneas harvested 2–6 days post infection (p.i.) for mRNA analysis. In Figure 1, CXCR2 was determined to be upregulated >240-fold above levels found in the controls at day 2 p.i. Upregulation of CXCR2 expression was significantly reduced by day 4 and 6 (84- and 21-fold, resp.). CXCR3 mRNA was observed to be most significantly upregulated at day 6 p.i with an expression level >28-fold higher than that of the uninfected control. This coincides with a >39-fold upregulation in the expression of the CD8 marker on infiltrating cells (data not shown). These results indicate that a marker for neutrophil recruitment (CXCR2) peaks in the cornea at day 2 p.i. with HSV-1 and that CXCR3 is present in the infected cornea at a time point associated with the recruitment of CD8^+^ T cells.

### 3.2. Expression of IP-10 mRNA Is Upregulated during the First 48 Hours of HSV-1 Infection of the Murine Cornea

**Figure 2:**
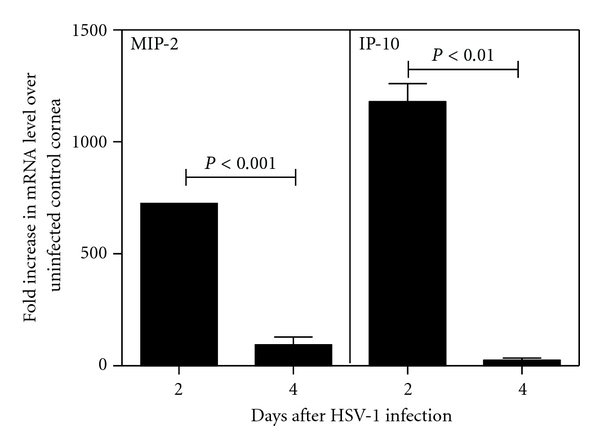
Expression of IP-10 mRNA is upregulated during the first 48 hours of HSV-1 infection of the cornea. Mice (*n* = 3) were challenged by intrastromal injection with 1 × 10^4^ PFU HSV-1 and corneas were harvested at the indicated times post infection. Control mice were uninfected and corneas from this group represented the level of constitutive mRNA production for each chemokine. Total RNA was isolated from each group and converted to cDNA which was analyzed by real-time PCR. Upregulation in mRNA for each chemokine is reported as the fold increase over production in uninfected control corneas.

 Having demonstrated the time points for expression of a neutrophil receptor and T-cell receptor in the model, experiments proceeded to investigate the potential to produce IP-10 in a time frame appropriate for neutrophils to be the source for this T-cell-recruiting chemokine.  Figure 2 shows that levels for upregulation of the mRNA for MIP-2 were found to be elevated >724-fold at day 2 p.i. dropping to >93-fold at day 4 p.i. Expression levels for IP-10 mRNA were highly upregulated (1179-fold over control) at day 2 after infection but the level of upregulation was reduced significantly by day 4 after infection (decreased to 24-fold over control). Thus, IP-10 message was found at the same time point as the peak in CXCR2 and MIP-2 mRNA expression which marked the infiltration of neutrophils. In addition, IP-10 mRNA expression was observed to be elevated prior to the time point for infiltration of T cells as suggested by the presence of CXCR3 mRNA (day 6 p.i.) and CD8a and CD8b mRNA (data not shown).

### 3.3. IP-10 is Produced at Significant Levels in the HSV-1 Infected Murine Cornea

**Figure 3:**
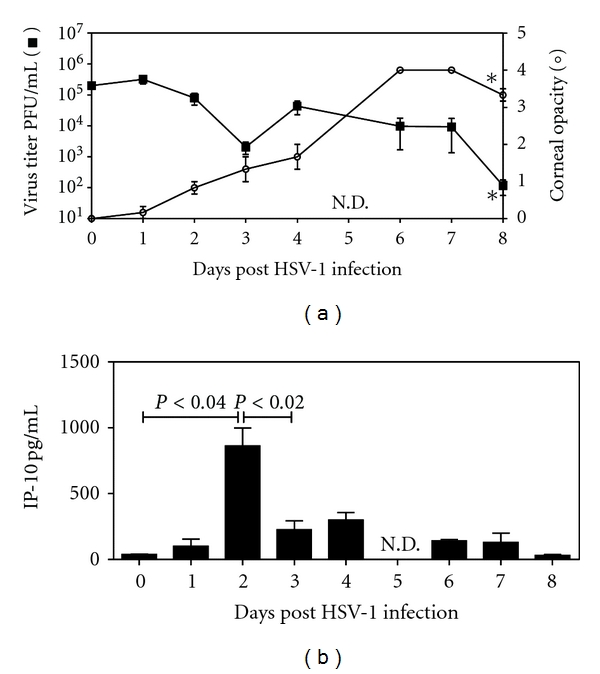
IP-10 is produced at significant levels in the HSV-1 infected cornea. Mice were infected with 2 × 10^5^ PFU HSV-1 on the scarified cornea. Mice were monitored for corneal opacity (graph (a), open circle, ∘) an asterisk indicates a significant increase between day 1 and 8. At the indicated time points corneas (*n* = 3) were excised and lysates produced for virus titration (graph (a), closed box, ■), an asterisk indicates a significant reduction from day 1 to day 8 (*t*-test) and the medians for the data are found to be significantly different by a nonparametric test (Kruskal-Wallis test). Levels of IP-10 protein production within the infected corneal lysates are shown in (b). N.D. = not done.

 Although chemokine and receptor mRNA expression profiles provided supporting evidence for the hypothesis that neutrophils may act as a cellular source for the T-cell-recruiting chemokine IP-10, it was still necessary to demonstrate the presence of the IP-10 protein within the model. The kinetics of IP-10 production were determined over an 8 day period after topical infection of corneas with 2 × 10^5^ PFU HSV-1. At each time point indicated on Figure 3, mice were monitored for corneal opacity as an indicator of the level of inflammation (Figure 3(a), ■). Mice were then sacrificed and corneal lysates produced to determine virus titers by plaque assay (Figure 3(a), ∘). IP-10 protein levels were quantitated by ELISA and are shown in Figure 3(b).

Figure 3(a) demonstrates that viral load (■) was significantly reduced by day 8 p.i. compared to the day 0 inoculum and that corneal opacity due to cellular infiltration was significantly increased (∘) in the same time frame. Constitutive expression of IP-10 was observed to be >38.6 pg/mL ± 2.8 SEM at day 0 in the model. The kinetics of IP-10 production are marked by a significant peak of 861.8 pg/mL ± 136.2 SEM at day 2 after infection IP-10 levels remain significantly high until day 7 and return to constitutive levels at day 8 p.i. (130.4 pg/mL ± 70.2 SEM and 30.1 pg/mL ± 7.6 SEM, resp.). This day 2 p.i. time point for peak IP-10 protein production agrees with that observed for peak IP-10 mRNA and peak CXCR2 mRNA expression.

### 3.4. Cells Resident in the Murine Cornea Have the Potential to Produce IP-10

**Figure 4:**
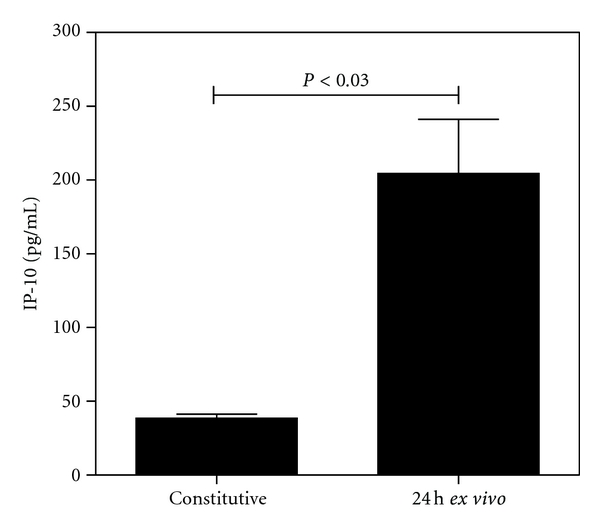
Cells resident in the cornea have the potential to produce IP-10. Corneas (*n* = 4) were excised from uninfected mice and placed in 250 *μ*L of serum-free medium. Corneas were either processed immediately for analysis of chemokine production (constitutive levels) or incubated at 37°C in 5% CO_2_ for 24 h. Chemokine levels were quantitated by ELISA and reported as pg/mL of sample.

 Murine corneas have been reported to be productive for mRNA for various chemokines including MIP-2 and IP-10 in a Balb/c HSV-1 infection model [25, 26]. As reported by Lundberg et al. the effects of IP-10 signaling may be strain specific [27]. Having established that IP-10 protein is produced at high levels within the C57 Bl/6 HSV-1 infected cornea, experiments were initiated to determine the potential of resident corneal cells from this strain of mouse to act as a source of IP-10. Mouse corneas were excised from uninfected hosts and incubated *ex vivo* for 24 h. Uninfected corneas were devoid of lymphocytes normally recruited during the inflammatory response to HSV-1. Constitutive levels of IP-10 protein were measured from samples which were excised and processed immediately. In Figure 4, it can be observed that constitutive levels of IP-10 were low (38.6 pg/mL ± 2.8 SEM) but that incubation for 24 hours led to a >5-fold increase in IP-10 levels. This data demonstrates that resident cells of the cornea have the potential to secrete IP-10 protein in response to excision trauma and may, therefore, contribute to the production of the chemokine during virus infection in the C57 Bl/6 model.

### 3.5. Cultured Murine Fibroblast Cells Produce High Levels of IP-10 in Response to Stimulation with Proinflammatory Mediators

**Figure 5:**
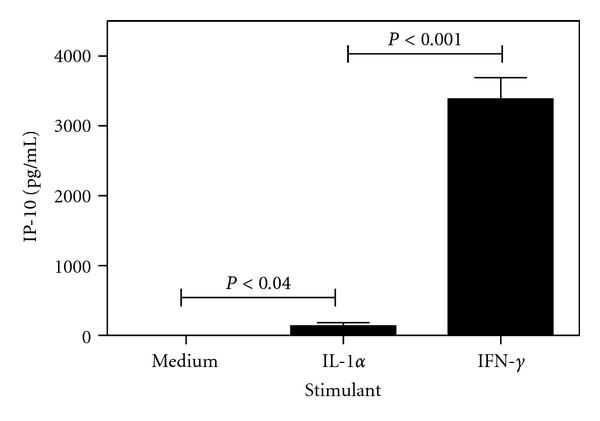
Cultured fibroblast cells produce IP-10 in response to stimulation with proinflammatory mediators. 4 × 10^4^ cultured mouse corneal fibroblast cells were incubated at 37°C and 5% CO_2_ in 500 *μ*L serum-free media with or without cytokine stimulation for 24 h. Cells were stimulated with 10 ng/mL of either recombinant mouse IL-*α* or IFN-*γ* and assayed for IP-10 production by ELISA.

 Tissue culture experiments were performed to further explore the potential for production of IP-10 by resident corneal cells as opposed to infiltrating leukocytes. Corneas from uninfected mice were excised and processed, as described in the materials and methods, to establish a primary corneal fibroblast cell line. 4 × 10^4^ fibroblast cells were plated under serum-starved conditions and stimulated with the proinflammatory cytokines IL-1*α* or IFN-*γ* at 10 ng/mL for 24 h. The cell supernatants were assayed and levels of IP-10 production are shown in Figure 5. Within the time frame of the stimulation, expansion of the cell population was not observed to occur (data not shown). Fibroblast cells produced high levels of IP-10 after stimulation with IFN-*γ* (3383 pg/mL ± 306 SEM). Although IL-1*α* stimulation also lead to significant IP-10 production compared to the media control, the level was >24-fold lower than that observed for IFN-*γ* stimulation. The ability of murine corneal fibroblast cells to produce IP-10 in response to stimulation with proinflammatory mediators was confirmed.

### 3.6. Purified Murine Neutrophils Produce High Levels of IP-10 in Response to Stimulation with Proinflammatory Mediators or HSV-1

**Figure 6:**
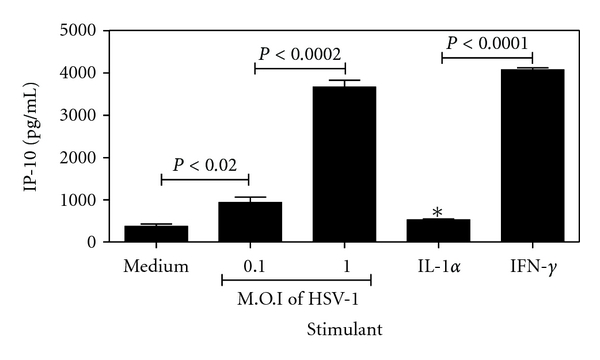
Purified mouse neutrophils produce high levels of IP-10 in response to stimulation with proinflammatory mediators or HSV-1. 1 × 10^6^ neutrophils purified from bone marrow were stimulated for 8 h *in vitro* with 10 ng/mL of either recombinant mouse IL-1*α*, IFN-*γ*, or HSV-1 at the dose indicated. Chemokine production was assayed by ELISA. An asterisk indicates a value not significantly different from the unstimulated control.

 Neutrophils are rapidly recruited to the HSV-1 infected cornea and are present in high numbers at 2 day after infection [4]. We observe this to also be the time point for peak IP-10 production in the model. We performed *in vitro* stimulation of bone marrow-derived neutrophils with IFN-*γ*, IL-1*α* or HSV-1 in order to determine the neutrophils potential to secrete IP-10.  Figure 6 illustrates that neutrophils produce high levels of IP-10 (4068 pg/mL ± 50 SEM) after incubation with 10 ng/mL IFN-*γ* for 8 h but did not produce significant levels of IP-10 after stimulation with the same concentrations of IL-1*α*. Neutrophils also responded to the presence of HSV-1 at a multiplicity of infection (M.O.I) of 0.1 by producing 937 pg/mL ± 126.5 SEM IP-10. A 10-fold increase in number of virus particles led to a 3.9-fold increase in chemokine production. Thus, neutrophils were demonstrated to have the ability to produce IP-10 in response to both proinflammatory cytokines and the presence of HSV-1. These results indicate a potential for neutrophils to contribute to the production of IP-10 within the HSV-1 infected cornea.

### 3.7. Neutrophil or NK Cell Depletion Lead to Significant Reduction of IP-10 Levels in the HSV-1 Infected Murine Cornea

 Having established that neutrophils were a potential cellular source of IP-10 *in vitro,* we performed *in vivo* depletion of these cells to determine their contribution to chemokine production during a primary HSV-1 corneal infection. NK and CD4^+^ T cells infiltrate the HSV-1-infected cornea [9] and have the potential to secrete cytokines including IFN-*γ* which could in turn stimulate neutrophils to secrete IP-10. We therefore included depletion of NK and CD4^+^ cells in this investigation. Peripheral neutrophil depletion was assessed by differential staining of tail vein blood smears to be reduced by >84% of the cell number observed in the IgG-control-treated mice. CD4^+^ T-cell and NK-cell depletions were monitored by FACS analysis of spleen cells and cell numbers determined to be reduced by >89% and >67%, respectively, when compared to the IgG control.

**Figure 7:**
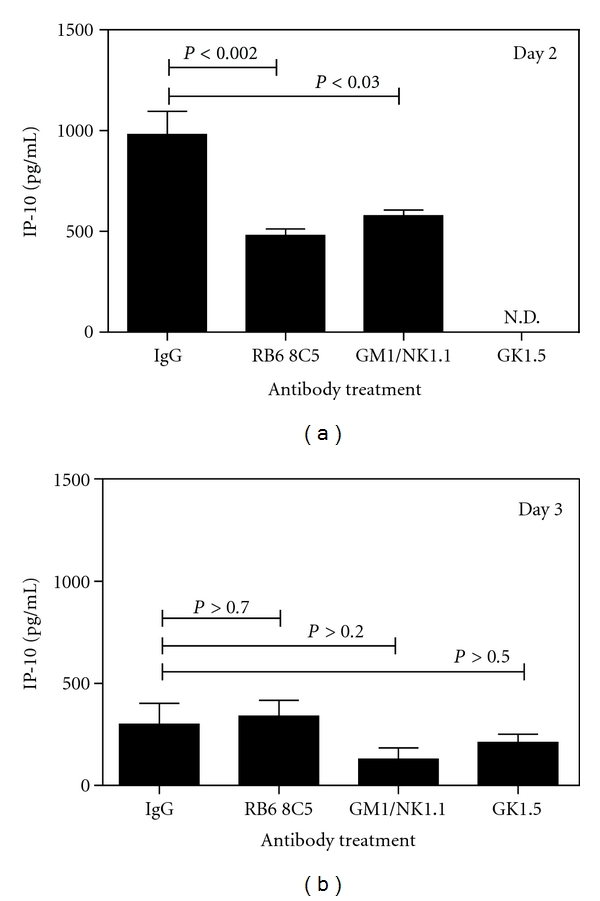
Neutrophil and NK depletion lead to a significant reduction of IP-10 in the HSV-1-infected cornea. Different groups of mice (*n* = 4) were depleted of each subset of cells by intraperitoneal injection 1 mg mAb RB6 8C5 (neutrophils), 1 mg antiasialo GM1 antibody admixed with 0.1 mg mAb, NK1.1 (NK cells) or 0.5 mg GK1.5 antibody (CD4^+^ cells) three hours prior to intrastromal injection of 1 × 10^5^ PFU HSV-1. Control animals received intraperitoneal injection of rat IgG prior to virus infection at the same dose. At days 2 and 3 post infection corneas were collected and processed for chemokine analysis via ELISA. Levels of IP-10 protein production within the infected cornea are shown as pg/mL for each sample. Cellular depletions were confirmed as described in the materials and methods. N.D. = not done.

 Two and three days post infection, corneas were harvested and analyzed to determine the effect of cellular depletion on IP-10 production.  Figure 7 shows a representative experiment. Neutrophil depletion by antibody treatment with RB6 8C5 produced a significant 2-fold reduction in IP-10 protein levels when compared to the IgG group on day 2 p.i. By day 3 p.i. IP-10 levels in the control corneas were greatly reduced (299 pg/mL ± 103 SEM) when compared to those observed at day 2 p.i. (931.4 pg/mL ± 111 SEM), and the effect of neutrophil depletion was abrogated. NK depletion also led to a 2-fold reduction in IP-10 levels at day 2 p.i. compared to the control. At day 3 p.i. the levels of IP-10 in the NK cell depletion group were not significantly different from that of the control. CD4^+^ T-cell depletion was not conducted at day 2 p.i. and does not significantly affect IP-10 production at day 3 p.i. in the model. These results indicate a role for both neutrophils and natural killer cells in the production of IP-10 in the model.

### 3.8. Corneal Cells Communicate with Neutrophils Leading to the Production of IP-10 during *Ex Vivo* Incubation of Human Corneas

**Figure 8:**
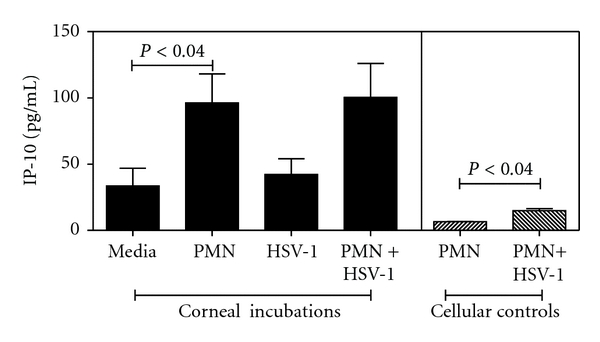
Corneal cells communicate with neutrophils leading to the production of IP-10 during *ex vivo* incubation of human corneas. 4 mm corneal buttons from human donor tissue were incubated in 500 *μ*L serum-free media at 37°C, 5% CO_2_ for 24 h. Where indicated corneal buttons were incubated in the presence of 1 × 10^6^ neutrophils and/or HSV-1 at a M.O.I. = 1. Cell controls were included to monitor IP-10 production in the absence of corneal tissue. IP-10 levels are shown as pg/mL of sample.

 In order to relate the findings of the murine model to infection of human corneal tissue with HSV-1, a novel *ex vivo* model was designed using donated human corneas. As described in the material and methods, this model permits the interaction of resident corneal cells, HSV-1, and neutrophils to be studied in the absence of other recruited inflammatory leukocytes which may be present *in vivo*. Corneal tissue for each experiment was obtained from a minimum of three independent donors and sectioned into 4 mm buttons. Results are shown in Figure 8. Corneal buttons from a single cornea were placed in one of the 4 test groups: control incubation with media (media column), incubation with 1 × 10^6^ neutrophils (PMN column), infection with 1 × 10^6^ PFU HSV-1, a M.O.I. = 1 with respects to the PMN (HSV-1 column) or infection with HSV-1-plus incubation with neutrophils (PMN + HSV-1 column). Corneal buttons incubated in isolation produced 33.7 pg/mL IP-10 ± 13.3 SEM. Infection of corneal buttons with HSV-1 failed to significantly increase IP-10 levels above this control. Incubation of corneal buttons in the presence of neutrophils led to a 3-fold increase in the production of IP-10 (96.8 pg/mL ± 21.9 SEM). HSV-1-infected corneas incubated in the presence of neutrophils also produced significant levels of IP-10 (100.4 pg/mL ± 25.7 SEM). Neutrophils incubated in isolation under conditions equivalent to those of the corneal tests produced relatively low levels of IP-10 which were significantly elevated on addition of HSV-1. Levels of CXCL8 production were also monitored for each corneal button and found to be significantly higher in HSV-1-infected corneas compared to the uninfected control (data not shown). CXCL8 is a proinflammatory chemokine produced at high levels during HSV-1 infection of the murine and human cornea. It is responsible for the recruitment of the neutrophil to the site of infection and marks the development of an inflammatory response [4].

 Corneal infection with HSV-1 in the absence of neutrophils was thus determined to be insufficient for elevated production of IP-10 in this model. Levels of IP-10 were observed to be elevated when traumatized or HSV-1-infected corneas were incubated with neutrophils. These results indicate that cellular interaction between corneal cells and neutrophils contribute to IP-10 production in a novel *ex vivo* human model and are supportive of the findings described above for the mouse model.

## 4. Discussion

 It is known that both neutrophils and CD8^+^ T cells are key players in the inflammatory response to primary HSV-1 infection of the cornea and that CD8^+^ T cells are required for viral clearance [12, 13, 24]. Although it is reported that neutrophils are also involved in the process of viral clearance, their specific mode of action is not yet fully understood [13]. Work by Gasperini et al. has previously demonstrated that human neutrophils can be stimulated to produce IP-10 and that neutrophil secretions are potent chemoattractants for NK and Th1 cells [28]. The requirement for IP-10 and CXCR3 during T-cell recruitment and activation in various inflammatory models has been established with the use of CXCR3 knockout mice. A model for *Bordetella* respiratory infection reported IP-10 induction and CXCR3 expression early during the inflammatory response and reported reduced lymphocyte and NK recruitment when CXCR3 knockout mice were investigated [29]. The requirement for CXCR3 signaling for mobilization and activation of NK and CD8^+^ T cells and the requirement for IP-10 for viral clearance during HSV-1 and HSV-2 infections has also been described [30–33]. In addition, neutrophils have been identified as a cellular source for IP-10 during ulcerative colitis disease and are required to sustain CD8^+^ cell recruitment during acute myocarditis [34, 35]. We have previously published data which indicates that neutrophils contribute to production of IP-10 and recruitment of CD4^+^ T cells in an inflammatory model of delayed type hypersensitivity (DTH) to HSV-1 in the mouse [19]. We now present data which confirms IP-10 is produced in a model for corneal inflammation due to HSV-1 infection and that neutrophils are a source of this chemokine *in vivo.*

 To generate further evidence linking the involvement of neutrophils in T-cell recruitment to the HSV-1-infected cornea, a time line for cellular marker and chemokine expression was generated. Due to the technical difficulties associated with the isolation and quantitation of cells infiltrating the cornea, we chose to use a semiquantitative real-time PCR screening assay to monitor receptor expression within the murine model. We established that the assay was predictive for cellular infiltration by confirming that CD3 and CD8 mRNA (T cell markers) were significantly upregulated in corneas at 6 days p.i. (data not shown). Continued screening demonstrated upregulation of mRNA for both the chemokine IP-10 and its receptor, CXCR3, at day 2 and 6 p.i., respectively. Day 2 p.i. was further marked by upregulation of the MIP-2 chemokine receptor (CXCR2). CXCR2 is expressed on fibroblasts, melanomas, and neutrophils. Of these cell types, the neutrophil is known to be recruited to the site of HSV-1 infection in the cornea. These results may be interpreted to predict neutrophil recruitment at day 2 p.i. which coincides with IP-10 mRNA upregulation and that CXCR3 expressing cells infiltrate to peak numbers by day 6 p.i. These findings are in accordance with those of Araki-Sasaki et al. and Cook et al. who reported IP-10 and CXCR3 message upregulation in the corneas of mice infected with HSV-1 [36, 37]. Taken together, these results confirm that neutrophils are recruited within an appropriate time frame to be a cellular source for IP-10.

 Although previous research has begun to explore the role of the neutrophil in production of T-cell-recruiting chemokines at the site of inflammation due to viral infection, other cell types in the corneal model may contribute to IP-10 production. Studies on human corneal epithelial (HCE) and fibroblast (HCF) cells by our research group have described the ability of these cells to express IP-10 after stimulation with proinflammatory mediators such as IL-1*α* and IFN-*γ* [38, 39]. Corneal production of IP-10 during the inflammatory response to other viral infections such as respiratory syncytial virus has been established [40]. In the study presented here, we confirm that murine corneas behave in a similar manner and secrete IP-10 constitutively at low levels and significantly elevated levels in response to proinflammatory mediators such as IL-1*α* produced by physical trauma (e.g., excision of cornea for *ex vivo* incubation). Furthermore, we show that the murine corneal fibroblast cells in isolation produce high levels of IP-10 when stimulated with IFN-*γ* and to a lesser extent IL-1*α*. The cells resident in the cornea can thus be demonstrated to have the potential to contribute to IP-10 levels during infection of the cornea.

 We have previously demonstrated the ability of murine neutrophils to secrete IP-10 *in vitro* in response to stimulation with IFN-*γ* but not IL-1*α* [15]. Here we expand the study to investigate the ability of purified neutrophils to secret IP-10 after *in vitro* stimulation with HSV-1. Neutrophils produce increasing levels of IP-10 in response to HSV-1 stimulation in a dose-dependent manner. The level of IP-10 observed in the assay after stimulation with HSV-1 at a M.O.I of 1 were equivalent to those observed for stimulation with 10 ng/mL IFN-*γ*, a known signal for IP-10 production in other models [19].

 These *in vitro* stimulation experiments provide evidence that within the HSV-1-infected murine cornea there is potential for either resident corneal cells or infiltrating neutrophils to be a source of IP-10. To investigate the relative contribution to IP-10 production by resident corneal cells versus infiltrating neutrophils we conducted *in vivo* cellular depletion experiments. Antibody-mediated cellular depletion of neutrophils, natural killer cells (NK) or CD4^+^ T cells has been shown to be an effective method for the study of cellular function within inflammatory models [13, 21–23]. We have demonstrated previously that such depletion leads to a reduction in numbers of the cells recruited to the site of inflammation within a DTH model [19]. Here we describe how IP-10 production was significantly reduced in response to reduction in numbers of either neutrophil or NK cells from the host. NK cells are not reported to be a source of IP-10 but do express the receptor for the chemokine (CXCR3) [41]. This suggests that in our study the reduction in IP-10 production observed after NK cell depletion is due to an indirect effect. NK cells are a source of IFN-*γ* which we demonstrate here to be an effective stimulant for the production of IP-10 by neutrophils. Thus the reduction in IP-10 production after NK cell depletion may be explained in terms of a reduction in IFN-*γ* stimulation of neutrophils. This conclusion is supported by observations in an IFN-*γ* gene knockout mouse which demonstrate reduced control of virus replication during corneal infections with HSV-1 [42]. Although CD4^+^ T cells may also secrete IFN-*γ*, results from depletion of this cell type do not indicate that the CD4^+^ T cell is required for IP-10 production in the *in vivo* model within the time frame studied. The depletion studies thus support the hypothesis that neutrophils act as a major cellular source for IP-10 during HSV-1 infection of the cornea and that loss of IP-10 production due to neutrophil depletion cannot be compensated for by IP-10 production by resident corneal cells.

 In the final set of data presented we investigated IP-10 production within an *ex vivo* model for human corneal infection by HSV-1. Previous work from our research group has focused on the chemokine and cytokine expression patterns from cultured HCE and HCF cells. In this study, we chose to maintain the potential for communication between these cell types by using corneal buttons comprised of all cell layers normally present in the human cornea. Therefore, we anticipated that the human model would reflect the findings from the murine *in vivo* model. Furthermore, the novel *ex vivo* human model described was designed to permit the study of interactions between resident corneal cells and purified cell populations, such as the neutrophil, in isolation from other cell types normally recruited to the site of inflammation *in vivo*.

 In the human *ex vivo* model levels of IP-10 produced by corneas increase significantly when the cornea was incubated in the presence of neutrophils. An equivalent result was observed when the corneas were infected with HSV-1 and incubated with neutrophils. This data suggests that proinflammatory mediators secreted by resident corneal cells in response to either physical trauma (excision) or viral infection lead to neutrophil production of IP-10. Corneal cells are reported to produce a variety of cytokines during response to viral infection including IL-1*α*, IL-6, CXCL8, and low levels of IFN-*γ* [43]. Although we found that murine neutrophils secreted high levels of IP-10 due to exposure to HSV-1 (Figure 6) this was not observed to occur to the same extent when human neutrophils were incubated with HSV-1 (Figure 8). Variations between experimental conditions and cellular origin may account for this difference. However, the results from the human *ex vivo* assay do indicate that IP-10 production by neutrophils *in vivo* is stimulated in part by communication from the cornea rather than as a direct response to neutrophil contact with the virus. It should be noted that in the *ex vivo* human model availability of virus for direct contact with the neutrophil is limited when the virus infects the corneal cells (viral eclipse). In conclusion, we present here evidence that neutrophils are a key source of the T-cell-recruiting chemokine, IP-10, during HSV-1 infection of the cornea. Our results suggest that cells resident in the cornea produce proinflammatory mediators in response to HSV-1 infection which recruit and stimulate neutrophils to produce IP-10. The neutrophils thereby contribute to the production of IP-10 during the early time point after infection (day 2). We hypothesize that this IP-10 leads in part to recruitment of NK cells which promote the inflammatory cascade by further stimulation of neutrophils with IFN-*γ* to increase IP-10 levels. Peak levels of IP-10 may subsequently recruit T cells leading to viral clearance.

## References

[1] Liesegang TJ, Melton LJ, Daly PJ, Ilstrup DM (1989). Epidemiology of ocular herpes simplex: incidence in Rochester, Minn, 1950 through 1982. *Archives of Ophthalmology*.

[2] Staats HF, Lausch RN (1993). Cytokine expression in vivo during murine herpetic stromal keratitis: effect of protective antibody therapy. *Journal of Immunology*.

[3] Lausch RN, Chen SH, Tumpey TM, Su YH, Oakes JE (1996). Early cytokine synthesis in the excised mouse cornea. *Journal of Interferon and Cytokine Research*.

[4] Yan XT, Tumpey TM, Kunkel SL, Oakes JE, Lausch RN (1998). Role of MBP-2 in neutrophil migration and tissue injury in the herpes simplex virus-1-infected cornea. *Investigative Ophthalmology and Visual Science*.

[5] Miyazaki D, Inoue Y, Araki-Sasaki K, Shimomura Y, Tano Y, Hayashi K (1998). Neutrophil chemotaxis induced by corneal epithelial cells after herpes simplex virus type 1 infection. *Current Eye Research*.

[6] Chen W, Tang Q, Hendricks RL (1996). Ex vivo model of leukocyte migration into herpes simplex virus-infected mouse corneas. *Journal of Leukocyte Biology*.

[7] Meyers-Elliott RH, Chitjian PA (1981). Immunopathogenesis of corneal inflammation in herpes simplex virus stromal keratitis: role of the polymorphonuclear leukocyte. *Investigative Ophthalmology and Visual Science*.

[8] Stumpf TH, Case R, Shimeld C, Easty DL, Hill TJ (2002). Primary herpes simplex virus type 1 infection of the eye triggers similar immune responses in the cornea and the skin of the eyelids. *Journal of General Virology*.

[9] Inoue T, Inoue Y, Kosaki R (2001). Immunohistological study of infiltrated cells and cytokines in murine herpetic keratitis. *Acta Ophthalmologica Scandinavica*.

[10] Stuart PM, Summers B, Morris JE, Morrison LA, Leib DA (2004). CD8^+^ T cells control corneal disease following ocular infection with herpes simplex virus type 1. *Journal of General Virology*.

[11] Suvas S, Kumaraguru U, Pack CD, Lee S, Rouse BT (2003). CD4^+^CD25^+^ T cells regulate virus-specific primary and memory CD8^+^ T cell responses. *Journal of Experimental Medicine*.

[12] Thomas J, Gangappa S, Kanangat S, Rouse BT (1997). On the essential involvement of neutrophils in the immunopathologic disease: herpetic stromal keratitis. *Journal of Immunology*.

[13] Tumpey TM, Chen SH, Oakes JE, Lausch RN (1996). Neutrophil-mediated suppression of virus replication after herpes simplex virus type 1 infection of the murine cornea. *Journal of Virology*.

[14] Divito SJ, Hendricks RL (2008). Activated inflammatory infiltrate in HSV-1-infected corneas without herpes stromal keratitis. *Investigative Ophthalmology and Visual Science*.

[15] Molesworth-Kenyon SJ, Oakes JE, Lausch RN (2005). A novel role for neutrophils as a source of T cell-recruiting chemokines IP-10 and Mig during the DTH response to HSV-1 antigen. *Journal of Leukocyte Biology*.

[16] Cassatella MA, Gasperini S, Calzetti F, Bertagnin A, Luster AD, McDonald PP (1997). Regulated production of the interferon-*γ*-inducible protein-l0 (IP-10) chemokine by human neutrophils. *European Journal of Immunology*.

[17] Farber JM (1997). Mig and IP-10: CXC chemokines that target lymphocytes. *Journal of Leukocyte Biology*.

[18] Carr DJJ, Chodosh J, Ash J, Lane TE (2003). Effect of anti-CXCL10 monoclonal antibody on herpes simplex virus type 1 keratitis and retinal infection. *Journal of Virology*.

[19] Molesworth-Kenyon S, Mates A, Yin R, Strieter R, Oakes J, Lausch R (2005). CXCR3, IP-10, and Mig are required for CD4^+^ T cell recruitment during the DTH response to HSV-1 yet are independent of the mechanism for viral clearance. *Virology*.

[20] Tumpey TM, Fenton R, Molesworth-Kenyon S, Oakes JE, Lausch RN (2002). Role for macrophage inflammatory protein 2 (MIP-2), MIP-1*α*, and interleukin-1*α* in the delayed-type hypersensitivity response to viral antigen. *Journal of Virology*.

[21] Daley JM, Thomay AA, Connolly MD, Reichner JS, Albina JE (2008). Use of Ly6G-specific monoclonal antibody to deplete neutrophils in mice. *Journal of Leukocyte Biology*.

[22] Rice JC, Bucy RP (1995). Differences in the degree of depletion, rate of recovery, and the preferential elimination of naive CD4^+^ T cells by anti-CD4 monoclonal antibody (GK1.5) in young and aged mice. *Journal of Immunology*.

[23] Welsh RM, Dundon PL, Eynon EE, Brubaker JO, Koo GC, O’Donnell CL (1990). Demonstration of the antiviral role of natural killer cells in vivo with a natural killer cell-specific monoclonal antibody (NK 1.1). *Natural Immunity and Cell Growth Regulation*.

[24] Russell RG, Nasisse MP, Larsen HS, Rouse BT (1984). Role of T-lymphocytes in the pathogenesis of herpetic stromal keratitis. *Investigative Ophthalmology and Visual Science*.

[25] Su YH, Yan XT, Oakes JE, Lausch RN (1996). Protective antibody therapy is associated with reduced chemokine transcripts in herpes simplex virus type 1 corneal infection. *Journal of Virology*.

[26] Tumpey TM, Cheng H, Yan XT, Oakes JE, Lausch RN (1998). Chemokine synthesis in the HSV-1-infected cornea and its suppression by interleukin-10. *Journal of Leukocyte Biology*.

[27] Lundberg P, Openshaw H, Wang M, Yang HJ, Cantin E (2007). Effects of CXCR3 signaling on development of fatal encephalitis and corneal and periocular skin disease in HSV-infected mice are mouse-strain dependent. *Investigative Ophthalmology and Visual Science*.

[28] Gasperini S, Marchi M, Calzetti F (1999). Gene expression and production of the monokine induced by IFN-*γ* (MIG), IFN-inducible T cell *α* chemoattractant (I-TAC), and IFN-*γ*-inducible protein-10 (IP-10) chemokines by human neutrophils. *Journal of Immunology*.

[29] Widney DP, Hu Y, Foreman-Wykert AK (2005). CXCR3 and its ligands participate in the host response to Bordetella bronchiseptica infection of the mouse respiratory tract but are not required for clearance of bacteria from the lung. *Infection and Immunity*.

[30] Thapa M, Carr DJJ (2009). CXCR3 deficiency increases susceptibility to genital herpes simplex virus type 2 infection: uncoupling of CD8^+^ T-cell effector function but not migration. *Journal of Virology*.

[31] Wuest TR, Carr DJ (2008). Dysregulation of CXCR3 signaling due to CXCL10 deficiency impairs the antiviral response to herpes simplex virus 1 infection. *Journal of Immunology*.

[32] Wuest T, Farber J, Luster A, Carr DJJ (2006). CD4^+^ T cell migration into the cornea is reduced in CXCL9 deficient but not CXCL10 deficient mice following herpes simplex virus type 1 infection. *Cellular Immunology*.

[33] Carr DJJ, Wuest T, Ash J (2008). An increase in herpes simplex virus type 1 in the anterior segment of the eye is linked to a deficiency in NK cell infiltration in mice deficient in CXCR3. *Journal of Interferon and Cytokine Research*.

[34] Noguchi A, Watanabe K, Narumi S (2007). The production of interferon-*γ*-inducible protein 10 by granulocytes and monocytes is associated with ulcerative colitis disease activity. *Journal of Gastroenterology*.

[35] Grabie N, Hsieh DT, Buono C (2003). Neutrophils sustain pathogenic CD8^+^ T cell responses in the heart. *American Journal of Pathology*.

[36] Araki-Sasaki K, Tanaka T, Ebisuno Y (2006). Dynamic expression of chemokines and the infiltration of inflammatory cells in the HSV-infected cornea and its associated tissues. *Ocular Immunology and Inflammation*.

[37] Cook WJ, Kramer MF, Walker RM (2004). Persistent expression of chemokine and chemokine receptor RNAs at primary and latent sites of herpes simplex virus 1 infection. *Virology Journal*.

[38] McInnis KA, Britain A, Lausch RN, Oakes JE (2005). Synthesis of *α*-chemokines IP-10, I-TAC, and MIG are differentially regulated in human corneal keratocytes. *Investigative Ophthalmology and Visual Science*.

[39] McInnis KA, Britain A, Lausch RN, Oakes JE (2007). Human corneal epithelial cells synthesize ELR-*α*-chemokines in response to proinflammatory mediators. *Ocular Immunology and Inflammation*.

[40] Bitko V, Garmon NE, Cao T (2004). Activation of cytokines and NF-kappa B in corneal epithelial cells infected by respiratory syncytial virus: potential relevance in ocular inflammation and respiratory infection. *BMC Microbiology*.

[41] Groom JR, Luster AD (2011). CXCR3 ligands: redundant, collaborative and antagonistic functions. *Immunology and Cell Biology*.

[42] Keadle TL, Alexander DE, Leib DA, Stuart PM (2008). Interferon gamma is not required for recurrent herpetic stromal keratitis. *Virology*.

[43] Biswas PS, Rouse BT (2005). Early events in HSV keratitis—setting the stage for a blinding disease. *Microbes and Infection*.

